# Reconsidering the Complex Role of Inflammation in Sudden Infant Death Syndrome

**DOI:** 10.7759/cureus.112019

**Published:** 2026-07-03

**Authors:** Paul N Goldwater

**Affiliations:** 1 Paediatrics, Adelaide Medical School, Adelaide University, Adelaide, AUS

**Keywords:** immunoparalysis, infection, inflammation, reactogenicity, sids, sudden infant death syndrome, vaccination

## Abstract

Within sudden infant death syndrome (SIDS) reside several primary phenomena. These include a state of immune immaturity, susceptibility to infection, and an inflammatory state. Most SIDS risk factors increase the risk of infection (prematurity, lack of breastfeeding, low or absent transplacental antibody, ethnicity, genetics, specific gene polymorphisms associated with susceptibility to infection, poverty, etc.). Most SIDS cases display evidence of an inflammatory state (raised inflammatory markers and inflammatory cytokines). The pattern of inflammation is very similar to that observed following vaccination. The complex relationship between reactogenicity and immunogenicity is examined. Under certain circumstances, reactogenicity can cause immune paralysis and may leave a vulnerable infant open to infection and systemic inflammatory response syndrome, leading to shock. This mechanism is explored in this rapid review in the context of the aetiopathogenesis of SIDS.

## Introduction and background

Sudden infant death syndrome (SIDS) is defined as the sudden, unexpected death of an apparently healthy infant under one year of age, which remains unexplained after a thorough case investigation, including a complete autopsy, examination of the death scene, and review of the clinical history [1]. It is important to recognize that SIDS is not only a diagnosis of exclusion but also a distinct entity within the broader category of sudden unexpected infant death (SUID) or sudden unexpected death in infancy (SUDI). SUID/SUDI includes those cases due to explained causes, such as accidental suffocation, metabolic disorders, or infections, as well as those that remain unexplained after investigation. SIDS specifically refers to those cases where no cause is identified after a comprehensive evaluation as per the above definition [2].

For many decades, there has been an unresolved problem in categorizing SIDS: the problem lies in the fact that a majority of SIDS cases bear features of infection [3-5], and the decision is problematic in naming these cases as SIDS or a death occurring in conjunction with infection that is not considered serious enough to have caused the death. Such deaths are therefore given the name SUID/SUDI. The ‘diagnosis’ depends on the individual perspectives held by the pathologist or coroner responsible for making it. Other papers provide perspective in this regard and emphasize infection-related features commonly observed in SIDS [6-8]. Infection-related consequences (including sepsis) are features of SIDS, and remain primary considerations, especially in the context of the much-relied-upon ‘triple risk hypothesis’ (in which a genetically or developmentally vulnerable infant at a critical developmental stage is exposed to exogenous stressors such as smoke exposure, prone sleeping, or overheating [9]. Infection and its accompanying inflammatory response are rarely mentioned but are accepted stressors. This generally downplayed role of infection (and associated inflammation) in SIDS pathogenesis may represent an important oversight in SIDS research.

Numerous studies on the role of infection in SIDS have been published [3-8,10-12]; however, this body of work has been generally ignored by mainstream researchers. This work has been consolidated in the last five years (vide infra). Current favored hypotheses regarding the pathophysiological mechanisms underlying SIDS are centred on the triple-risk model, which proposes that SIDS occurs when three factors converge: (1) an intrinsically vulnerable infant (often with genetic, neurodevelopmental, or metabolic abnormalities), (2) a critical developmental period (typically the first one to four months of life), and (3) exposure to exogenous stressors (such as prone sleep position or unsafe sleep environments) [1]. This model is endorsed by the American Academy of Pediatrics [13]. The hypothesis at the core of this paper centres on infection (and related phenomena), which also aligns with the triple-risk hypothesis (vide infra).

The review centres on potential immunopathological events arising from inflammatory processes (arising from infection and/or vaccination) that could contribute to SIDS causation, while acknowledging foundational tenets of the triple-risk model, given that the model is also applicable to most infectious diseases affecting infants. Vulnerability is associated with the stage of development and susceptibility to an environmental stressor (the infectious agent). While the review focuses on infection, inflammation, and vaccination as potential stressors, it still recognizes the importance of other triple-risk stressors, including prone sleep position, and household and antenatal smoke exposure, as these are likely to co-occur in instances of SIDS and are known to interact with infection. The immunopathological events could represent a tipping point for some cases to occur.

**Figure 1:**
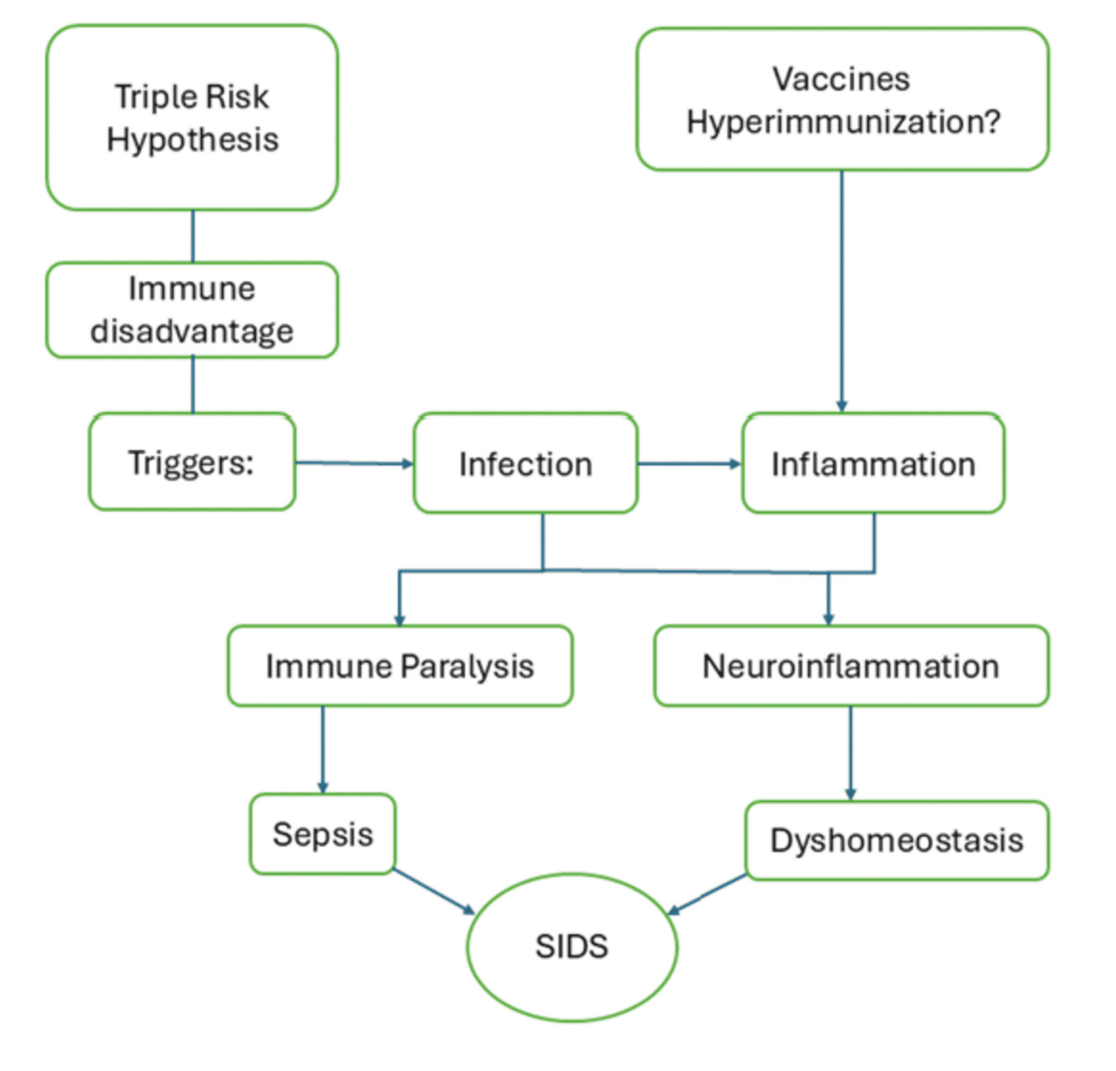
Graphical abstract displaying the key elements of the review SIDS: Sudden infant death syndrome. Image credit: Created by the author using Microsoft PowerPoint (Microsoft Corp., Redmond, WA, USA).

It also examines SIDS with respect to its several primary attributes, which include immune immaturity, susceptibility to infection, and an inflammatory state. Most SIDS risk factors are associated with a higher risk of infection (prematurity, lack of breastfeeding, low or absent transplacental antibody, ethnicity, genetics, specific gene polymorphisms associated with infection, poverty, etc.), and a majority of cases display evidence of an inflammatory state (raised inflammatory markers and inflammatory cytokines) with the pattern of inflammation sharing features observed following both infection and vaccination. It considers the complex relationship between reactogenicity (involving inflammation-related effects of vaccines) and immunogenicity (vaccine effectiveness), while reactogenicity, under certain circumstances, may lead to immune paralysis, which may leave a vulnerable infant open to infection and systemic inflammatory response syndrome, leading to shock. Thus, the scope of this review entails a broad examination of the mechanisms by which the effects of infection and inflammation could contribute to the aetiopathogenesis of SIDS, and addresses controversial aspects of vaccination in relation to the occurrence of SIDS (Figure 1).

The review utilized PubMed, Google Scholar, and Open Evidence to source relevant peer-reviewed literature using the search terms: sudden infant death syndrome; SIDS; SIDS and inflammation; SIDS and infection; SIDS and vaccination; Infants and Vaccination; Infants and Immunization; vaccine reactogenicity; infant immune paralysis; infant immunoparalysis; infant mortality. vaccine adverse effects. Abstracts were reviewed, and the papers included and read if relevant, which referred to studies in infancy. Case reports and papers solely involving children older than one year were not considered. Quality assessment tools were not used, given that the papers had been peer-reviewed. The hypothesis that infection is the most likely factor in the SIDS story, because of its strong association with most SIDS risk factors, and the diminished immunological status of the infant (lack of breastfeeding, prematurity leading to low maternal transplacental antibodies, etc.), representing “immune disadvantage,” and the consistent pathological finding of inflammation [4,6,14,15], stands as a well-supported idea.

The concentration by mainstream researchers on sleep and homeostatic control and arousal failure has produced little, if any, data of real substance despite decades of endeavor. The best support for a sleep-related homeostatic failure hypothesis is contained in the findings of CNS inflammation with microglial and astroglial activation, apoptosis, and raised levels of inflammatory cytokines (e.g., IL-6) in the cerebrospinal fluid (CSF). The recently published paper by Opdal et al. [16], entitled ‘The vicious spiral in Sudden Infant Death Syndrome’, provides a good summary of the present state of research but continues to invoke the triple risk hypothesis as the central tenet. The authors tended to favour CNS inflammatory responses as the key feature for the final pathway to death through disturbed homeostatic function [16] rather than seeing inflammation in a broader context.

Some further support for the infection model was shown by the increased rate of SIDS during the COVID-19 pandemic. This increase was most evident in 2021, with SIDS rates rising by ~10% compared to the pre-pandemic period, and a marked monthly increase observed from June to December 2021 (northern hemisphere) [17]. Additionally, the overall rate of SUID, which includes SIDS, plus cases resulting from an unsafe sleeping environment, and deaths of unknown cause, also increased during the active pandemic period, but the rise was primarily driven by SIDS rather than other SUID categories [17,18]. While pandemic-related factors associated with the increased rates of SIDS (such as disruptions in healthcare access, changes in sleep practices, especially co-sleeping), and altered patterns of respiratory infections, particularly off-season resurgences of respiratory syncytial virus (RSV) were main features and may add weight to the role of infection in SIDS [17,19,20]; on the contrary and in general, the COVID period was associated with marked decreases in other respiratory and gastroenterological infections which would be expected to be associated with a reduction in SIDS. The pandemic possibly influenced prenatal risk factors arising from parental behavior changes (increased smoking and drug taking) and effects of living in confinement. Substance use (including alcohol, nicotine, cannabis, and opioids) increased for a subset of adults during the COVID-19 pandemic, driven by stress and social disruption, although some studies reported stable or decreased use among others [17-21]. Maternal smoking and drug use are well-established, dose-dependent risk factors for SIDS, with risk further elevated by bed sharing and hazardous sleep environments. Combined use of alcohol and smoking during pregnancy also increases SIDS risk substantially [18,21].

The rise of proinflammatory cytokines in the CNS of SIDS cases are well known [16,20], and CNS viral infection is now recognized as part of the SIDS problem [22], confirming earlier studies. Perhaps the strongest evidence of infection playing a role in SIDS is the association of infection with prone sleep position [23,24] and this combined with Staphylococcus aureus infection carries a high relative risk of SIDS [25,26], while not overlooking the multitude of other infection-related factors listed in Table 1 [27-63].

**Table 1:** Risk factors relating to inflammation or infection SIDS: Sudden infant death sydrome.

Risk Factors relating to inflammation or infection
Genetic (Intrinsic) [27,28]
Gender: Possible X-linked genetic mutations/copy number variations, etc. innate and adaptive immunity, inflammatory response, nitric oxide synthetase 1 (NOS1), (flavin-monooxygenase 3, enzyme metabolising nicotine)
Brainstem function, Metabolic pathways, Cardiac function
NB. Infection and inflammation can affect these and cause dysfunction [16]
Ethnicity [29,30]
Extrinsic factors
Demographic factors [1,31]
Low socioeconomic status [32]
High birth order/previous live births [33]
Prenatal risks [34,35]
Inadequate/Poor prenatal care [36-38]
Maternal smoking/nicotine use [1]
Maternal misuse of drugs: Heroin, cocaine, and other drugs [1,39]
Subsequent births less than 1-year apart [40-42]
Maternal genitourinary infection [33]
Maternal Alcohol use [1,40]
Mother being overweight [43]
Teen pregnancy [44]
Maternal anemia (independent risk factor) [43,44]
Postnatal
Higher risk of unsafe sleeping environment [1,43]
Seasonality [45]
Viral respiratory or gastrointestinal symptoms in the days before death [46]
Sleeping prone with Staphylococcus aureus [25,26]
Prematurity [47]: increases risk of SIDS death by about 4X [48]
Low birth weight [33,47]
Exposure to tobacco smoke [1]: Immune suppression, enhancement of bacterial adherence and toxigenicity [7]
Room temperature: Elevated or reduced room temperature [1,48]
Excess bedding, clothing, soft sleep surface stuffed animals (fomites) [1,48]
Co-sleeping: Bed-sharing with parents or siblings [49]
Sofa-sleeping [50]
Sleeping on a used mattress [51]
Sleeping in parental bed [49]
Infant’s age: Incidence rises from zero at birth, highest from 2 to 4 months, declines toward zero at I year(peak SIDS coincides with nadir of maternally transferred antibodies) [7,29]
Probable anemia: (Hemoglobin hard to measure postmortem) [52]
Early cord clamping: Causing anemia or iron deficiency, obviates placental transfer of stem cells, immune cells and immunoglobulins [53-57]
Recent visit to general practitioner or outpatient clinic [58]
No or late immunizations [59.60]
Day care attendance [61]
Increased incidence of nighttime deaths: these are related to infection/ inflammation risk factors (illness and smoke exposure) [62,63]

## Review

Extending the infection hypothesis

This rapid review aims to seek additional putative factors that could explain the SIDS enigma. Given that inflammation is common in SIDS, the basis of which is partly explained by infections, which are very common in SIDS cases, the review raises a question raised in a previous paper by the author [64]: Is there or are there other potential causes of inflammation that play a role in the SIDS story? Vaccination fits this role. Vaccination seems to mimic most of the inflammatory properties (at the cellular and cytokine level) of targeted bacterial and viral infections. How this could impact infants in their first year of life is core to this discussion.

Vaccination/immunization: akin to infection

Obviously, where infection occurs, there is always an infectious agent (or agents-in-combination). Manufactured antigens contained in vaccines mimic actual natural infections to produce protective immune responses, but without most of the associated adverse effects of the infections.

Natural infectious diseases cause substantially higher rates of serious complications, hospitalizations, and deaths compared to vaccines, which typically produce only mild, self-limited adverse effects. The clinical differences span severity, duration, organ system involvement, and long-term sequelae.

In terms of severity and mortality, the fundamental clinical difference is that infectious diseases cause severe illness and death at rates orders of magnitude higher than vaccines. For example, for measles, natural infection causes death in approximately one per 1,000 cases (one to 15 per 100 in developing countries), while measles vaccination has zero documented deaths. Before polio vaccination, approximately 15,000-20,000 paralytic cases occurred annually, with 1,800-3,000 deaths; with vaccination, the incidence is near zero. For each licensed vaccine, the risk of adverse reactions is much lower than the risk of complications from the illness prevented, notwithstanding the issue of polio provocation through vaccine injection or tonsillectomy [65]. The above facts do not change the issue regarding the conjectural potential effects of vaccine-induced inflammation tipping the balance in favor of a SIDS outcome from vaccinations and associated adjuvants in single or multiple doses in immunologically, physiologically, and genetically vulnerable babies exposed to environmental stressors, e.g., smoke or acute respiratory viral infection. This conjecture is the focus of this review.

Until recently, there has been little consideration of vaccines, including attenuated real infections, in the context of SIDS. A recent paper raised the potential problem of hyperimmunization, wherein multiple and/or repeated antigenic exposure may cause serious, potentially fatal immunopathological responses [64]. Hyperimmunization is a term traditionally used in multiple or repeated immunizations to raise hyperimmune sera. For this review, the term is used in the context of multiple and/or repeated immunizations of human babies. It remains conjectural if these multiple exposures to vaccine antigens (in multiples at one time and repeats) and their associated adjuvants are potentially harmful to immunologically vulnerable babies exposed to environmental stressors, e.g., smoke or acute respiratory viral infection.

Studies cited in this review have shown increased infant mortality with an increasing number of vaccines received [66-68]. This forms the basis for generating a hypothesis, while its statistical limitations forbid attribution of causality. The 2012 study by Goldman and Miller [68] examined approximately 38,000 infants who were either hospitalized or died after receiving the scheduled vaccines. The researchers used data from the Vaccine Adverse Event Reporting System (VAERS) and grouped the cases resulting in serious adverse events by the number of vaccine doses received, which ranged from one to eight. Among the infants who received two doses of vaccine, the hospitalization rate was 11%; for three doses, it was 12.4%. The rise continued with each additional dose and reached 23.4% after eight doses. Infants who had five to eight doses had significantly higher mortality rates than those who received one to four doses.

VAERS data has limited application, given that it is collected passively and therefore represents only a tiny fraction of the number of deaths reported following vaccination, and categorization of causes is questionable. The value of these studies has been rightly questioned because of other limitations, including a lack of control for age, a primary confounding variable in pediatric vaccine research. The number of vaccine doses is intrinsically tied to an infant’s age, and older infants have accumulated more time to experience an adverse health event [69]. Moreover, the dose-dependent mortality suggested by Miller and Goodman could represent this temporal bias rather than a causal link. The question is: should these VAERS studies be ignored, or should they be regarded as small signals indicating a potentially true effect? To answer the latter, then, in the interests of future generations and the precautionary principle, it should be explored further. However, the number of vaccine doses routinely given in developed nations and their association with mortality rates provides a different and concerning perspective [70], as do more direct studies, which provide somewhat stronger evidence and may reflect problems with specific vaccines or adjuvants [71]. In contrast, numerous authors [72-79] have concluded that no vaccine-SIDS association exists. The studies discounting an association between vaccines and SIDS may also be flawed, but in different ways from the VAERS studies: in their design, including not covering all confounding factors.

The evidence for an association between vaccines and infant mortality must be regarded with great care, but it should raise a level of concern to warrant serious investigation. Many of the previous studies tended to use unadjusted analyses and lacked control for confounding variables, which is a significant methodological flaw in epidemiological research on vaccines evaluating risk of neurodevelopmental problems and mortality. However, to ignore these signals, however small, would be scientifically negligent when considering infant health and well-being in the context of the future of childhood immunization.

Methodological Issues

The main methodological problems in vaccine studies assessing the risk of infant mortality (and/or SIDS) from multiple vaccinations are summarized below, and include confounding, selection bias, misclassification, and limitations in study design.

Confounding is a major issue, particularly the "healthy vaccine effect," where infants who are vaccinated are generally healthier, and those with recent illness (who are at higher risk for SIDS) may have immunizations deferred. This can lead to an apparent protective effect of vaccination that is not causal [80]. Socioeconomic, maternal, and birth-related factors also confound the association, and not all studies adjust adequately for these variables [80].

Selection bias is incurred when cases and controls are not matched appropriately, or when unvaccinated infants are overrepresented among SIDS cases due to underlying health or social factors [81].

Misclassification of SIDS and timing of vaccination can also affect results, as SIDS peaks at ages when multiple vaccines are administered, leading to coincidental temporal associations [1]. The medical literature demonstrates that the overlap between the typical age window for SIDS (two to four months) and the recommended vaccination schedule can result in a non-causal clustering of SIDS cases shortly after vaccination, simply due to chance and age-related coincidence rather than causality [81]. Study design limitations include reliance on case-control studies, which are susceptible to recall and reporting bias, and ecological studies, wherein causality at the individual level cannot be established [80-84]. The use of passive surveillance systems like VAERS is limited by underreporting and lack of denominator data [83,84]. VAERS data are considered useful for signal detection and hypothesis generation, not for establishing causality or quantifying risk [82]. More robust designs, such as self-controlled case series, help address time-independent confounders but are less commonly used [81-83].

Single Versus Poly-vaccination

A recent meta-analysis by Boccalini et al. [85] elicited another “signal.” The study demonstrated that the administration of three or more injectable vaccines during a single visit is safe compared to controls vaccinated with three vaccines (three injections) in subsequent administrations, i.e., not in a single session.

However, significant systemic findings, including fever >38°C (OR 2.92, 95% CI 1.93-4.43), were observed with multiple vaccines given at one time, which points to increased reactogenicity and inflammatory response in this group. The study considered single poly-vaccination safe; however, there is concern regarding the possibility of increased inflammation underlying the pyrexia in some individuals.

While methodological challenges must be carefully considered when interpreting findings from vaccine-SIDS ​​​​​​studies, the American Academy of Pediatrics remains confident in interpreting the evidence as showing no causal relationship between immunizations and SIDS, but considers that observed protective effects may be partly attributable to confounding factors [1]. This exposes a possible inconsistency.

The problems in interpretation of the above and other studies were recently discussed by Cauchi et al. (2022) [82] in the context of vaccines and neurodevelopmental disorders. The study outcomes showed that determining causality was challenging, especially in understanding and accurately communicating the data to clinicians and the general population under so-called ‘infodemic’ conditions, facilitated by the electronic media.

Jacobson & Jacobson (2005) [86] provided a more detailed examination of the problems involved in study design and interpretation of data in the context of neurotoxins and neurodevelopmental disorders. They focused on multiple comparisons, effect modifiers, and clinical significance. Their approach was to avoid traditional statistical approaches because of inherent problems (including frequent under-powering) and favored methods that accounted for the marked differences in individual vulnerability that accompany most neurodevelopmental conditions or exposure risks, and recommended the use of effect modifier analysis and stratified subgroup approaches [86].

So, the risk of misinterpretation applies to studies both for and against a specific apparent correlation. Thus, it is now incumbent on vaccine manufacturers to conduct trials designed to overcome the inherent methodological problems and elevate the interpretation of results to a less controversial status. Conflict of interest remains a problem and should require manufacturers to have independent researchers formally involved in their trials.

As mentioned in the twin SIDS paper [64], the 2024 study by Jablonowski & Hooker [71] evaluated neurodevelopmental and other health outcomes among 1,542,076 vaccine combinations administered to infants younger than one year of age. The study raised concerns, particularly because it reported that sepsis developed in six of 227,231 infants who received Diphtheria, Tetanus, Pertussis acellular (DTPa)+inactivated polio vaccine (IPV)+Haemophilus influenzae type b (HIB), compared with 26 of 73,792 infants who received DTPa+IPV+HIB+PNC (pneumococcal)+hepatitis B (HepB)+rotavirus (Rota). This difference was statistically significant. The high risk of mortality from sepsis did not go unnoticed. While the study did not examine the risk of SIDS or SUDI, it showed that for each additional vaccine given, the number of diseases (neurodevelopmental, respiratory, or suspected infection) diagnosed more than doubled [71]. For example, the lack of physiological development significantly increased from 135 to 385 cases, and failure to thrive increased from 107 to 290 cases, while the opposite effect was observed for conditions including cough and asthma. Despite the methodological weakness of the study, it would be negligent if serious attention was not paid to this potentially adverse “dose effect,” possibly occurring through hyperimmunization. A crucial point made by Jablonowski et al. [71] was “though health safety agencies may claim safety of individual vaccines, claims to safety of vaccine combinations are unfounded.”

Hyperimmunization is a process of repeatedly exposing an individual to an antigen, which can have serious adverse effects (vide infra). Because hyperimmunization occurs with multiple immunizations, it is essential and necessary to determine if adverse effects of this hyperimmunization-like process could plausibly occur in infants who receive multiple and repeated immunizations via the infant immunization schedule [64]. If the data supports this, then gaining an understanding of how this could result in fatalities would be required. It is the purpose of this rapid review to discuss these mechanisms in as much detail as current knowledge allows.

Hyperimmunization: Vaccine Antigens or Adjuvants?

Firstly, what are the possible adverse outcomes observed following hyperimmunization? In 1902, Zenoni [87] first described the development of amyloidosis in horses hyperimmunized with the diphtheria toxin. Numerous other immunopathological outcomes are now well known to occur under similar excessive antigenic exposures. However, closer examination would indicate that the problem could reside in both the antigen(s) and the adjuvant(s). There is sufficient evidence to raise concern over multiple exposures to antigens [70,88-90] and multiple dosing of adjuvant(s), particularly those that are highly inflammatory [91,92].

According to Petrovsky [91], typical symptoms and signs of reactogenicity (fever, headache, malaise, nausea, diarrhea, arthralgia, myalgia, and lethargy are manifested through adjuvant-associated innate immune activation and inflammatory responses. Adjuvants are used to exaggerate the activation of innate immune receptors. Certain adjuvants function as pathogen-associated molecular patterns (PAMPs) and induce systemic reactogenicity. Toll-like receptor (TLR) ligands, such as monophosphoryl lipid A (MPL), flagellin, lipoarabinomannan, peptidoglycan, or acylated lipoprotein [92] are Petrovsky’s examples. Adjuvants such as oil emulsions and saponins cause tissue damage and produce damage-associated molecular patterns (DAMPs) that activate innate immune receptors and invoke inflammation [91-93] and consequent systemic reactogenicity.

The duration of inflammation-associated adjuvant reactogenicity (markers of inflammation/reactogenicity such as C-reactive protein (CRP, an acute-phase reactant) and interleukin-6 (IL-6)) typically rises within hours after immunization in infants and generally returns to baseline within 48-72 hours. In premature infants, elevations in CRP and IL-6 are evident after administration of whole-cell pertussis-containing vaccines, peaking within the first 24-48 hours and normalizing by 48-72 hours post-immunization [94,95]. However, transcriptomic changes, including overexpression of interferon-stimulated and inflammation-related genes, are most pronounced at seven days post-vaccination and generally resolve by one month after immunization in term infants [96].

Single Versus Poly-vaccination and CRP

It is important to clarify if there is any difference between the administration of a single vaccine or of multiple vaccines to infants on one occasion. Pourcyrous et al. [97] demonstrated that abnormal elevation of CRP level occurred in 85% of premature infants (<35 weeks of gestation) who had received multiple vaccines. By comparison, raised CRP occurred in up to 70% of those who received a single vaccine. Of note, in 16% of infants, cardiorespiratory events were observed within 48 hours postimmunization. These were deemed to be vaccine-associated by the authors.

Abnormal CRP values were associated with multiple vaccines (OR, 15.77; 95% CI 5.10-48.77) and severe intraventricular hemorrhage (IVH) (OR, 2.28; 95% CI 1.02-5.13) in logistic regression analysis. Cardiorespiratory events were associated marginally with receipt of multiple injections (OR, 3.62; 95% CI 0.99-13.25) and significantly with gastroesophageal reflux (GER) (OR, 4.76; 95% CI 1.22-18.52). The vaccines used included DTaP, Hib (ActHIB), HBV (Engerix-B), IPV (Inactivated-IPOLTM), and 7-valent pneumococcal conjugate vaccine (PCV7 or Prevnar). Infants were assigned to one of five vaccines, each with different adjuvants, making it challenging to interpret combined results. However, it was clear that DTaP caused the highest average CRP (12.1 mg/dL), the next highest being ActHIB (7.1 mg/dL). DTaP incorporates alum as its adjuvant. The adjuvant in ActHIB is tetanus protein (PRP-T) conjugated to Hib capsular polysaccharide (PRP). When all these vaccines were given on the same day to infants, the average CRP was the second-highest recorded overall (10.8 mg/dL). The study showed that premature babies admitted to NICU given DTaP are apt to experience cardiorespiratory adverse events and elevation of CRP. Natural infection induces an acute phase reaction through the actions of the cytokines IL-1β and IL-6, and liver-derived CRP is detectable within six hours and is maximal at 48h post-infection [94-97]. CRP has been found to remain elevated for up to six days after death [98,99].

When comparing multiple vaccines with a single vaccine, higher rates of adverse events (in addition to CRP) are observed after administration of the former compared to the latter [97]. It is interesting that in the context of SIDS, compared to infection, the inflammatory markers of IL-1 and CRP seemed to be higher (on average) in the infection group than in SIDS, but there was no difference between the two groups in 17 other (mostly pro-inflammatory) immune biomarkers [100]. In febrile infants younger than three months, post-immunization leukocytosis was observed in almost half the children. These cases met the criteria for formal sepsis workup, yet they had no clinical symptoms or signs of infection [101].

Many infants are iron-deficient during the months when the risk of SIDS is highest. Such hypoferronemia can have significant effects on the immune response to both infections and vaccinations [102]. The underlying mechanisms are complex and will be discussed in a separate paper.

Comparing SIDS with infectious causes

If SIDS (or a subgroup thereof) is somehow the result of an infection or vaccination, then we should question the idea of comparing SIDS with defined infections. As we have seen, it is difficult to separate the two based on blood levels of a long list of pro-inflammatory markers. 

However, the findings in relation to vaccine reactogenicity only strengthen the notion that immunization (especially involving adjuvants) mimics infection in terms of the pro-inflammatory and reactogenic molecules produced.

In summary, post-vaccination inflammatory markers develop in the first few days post-injection and resolve in most recipients within a week, and can persist for a month. There is a suggestion that the degree of reactogenicity is related to the adjuvant used, and most importantly, the timing of the reactogenicity coincides with the incidence of unexpected infant deaths reported following immunization (summarized in Goldwater 2025 [64]), with higher mortality in the week following immunization compared to mortality at eight weeks post-immunization, just before the next series of vaccines is scheduled.

Von Kries et al. [103] assessed whether temporal associations of mortalities could be attributed to chance. Their study used standardized mortality ratios (SMR) for deaths occurring within 28 days following either of two hexavalent vaccines in the first or second year of infants. The authors determined SMRs by using respective annual rates for sudden unexpected deaths (SUDs) from national vital statistics. With one of the vaccines, there was an insignificant increase in SMR in those under one year of age, while the other vaccine was associated with a significant increase in SMR on days one and two of vaccination in one- to two-year-olds. The authors were unable to explain the apparent vaccine effect in the older group [103]. They concluded that the findings, based on spontaneous reporting, did not prove a causal relationship between vaccination and SUDs. However, the data constituted a “signal” for one of the two hexavalent vaccines examined, which requires ongoing monitoring.

The report by Zinka et al. [104] described five infants who died of apparent SIDS within 48 hours of receiving hexavalent vaccines. Their ages fell within the typical peak SIDS range (two to four months). While causation cannot be firmly attributed to the vaccine, signs of severe reactogenicity in three infants who were febrile (up to 39^◦^C) were of note. Such reported clinical features, especially high fever, indicate that inflammation, through the release of pro-inflammatory cytokines, is likely involved as a result of the vaccine’s reactogenic effects, assuming other causes were excluded.

Role of inflammation

To achieve successful immunization, inflammation (which occurs with natural infection) is essential for achieving protective immunity, as it facilitates the development of adaptive immunity by activating innate immune pathways and promoting cytokine and chemokine production [105,106]. This local and systemic inflammatory response is typically transient and manifests as common, mild adverse effects such as pain, erythema, swelling at the injection site, and fever or malaise [107-109]. These symptoms are generally self-limiting and correspond with increased levels of proinflammatory cytokines (e.g., IL-1β, IL-6, tumor necrosis factor-alpha (TNF-α), granulocyte colony-stimulating factor (G-CSF)) and seem to be more pronounced with adjuvanted vaccines [110-111].

Inflammatory Adverse Events

In a minority of cases, inflammation can lead to more significant adverse events, including febrile seizures, arthralgia, arthritis, cutaneous reactions, and, rarely, vascular leak syndrome and immune-mediated phenomena such as uveitis, idiopathic thrombocytopenic purpura, or acute disseminated encephalomyelitis [112,113]. Reports that mRNA COVID-19 vaccination exacerbates or induces immune-mediated inflammatory diseases are rare [114]. Causality has not been established [114,115]. In a small study by Tukaj et al. [115], the COVID-19 mRNA vaccine did not affect major T-helper cell cytokine levels, including Th1, Th2, Th17, and Th22. These cytokines are central to the differentiation and effector functions of their respective T-helper cell subsets and are implicated in autoimmune pathogenesis [115]. However, anti-Hsp (heat shock) autoantibodies, which are associated with autoimmune diseases, were shown to be restricted to mRNA vaccine recipients and were not found in unvaccinated and never-infected controls. This finding should raise concern and require serious investigation [116]. Clinically severe reactogenicity of mRNA vaccines that utilize encapsulated lipid nanoparticles (LNPs) was recently attributed to the LNPs rather than to the vaccine's mRNA content [117] based on a murine model.

Neonatal bloodspot findings

A 2024 study by Oltman et al. [118] associated postnatal measurements of blood metabolites in neonatal bloodspots with later development of SIDS. The authors showed an association with elevated acylcarnitine levels, particularly free carnitine and C-14OH (3-hydroxytetradecanoylcarnitine), which were linked to a higher risk of SIDS. The former crosses the placenta, whereas the latter does not, thus inferring C-14OH was of fetal origin. The findings are of special interest given that these molecules, particularly long-chain species, are produced as part of the inflammatory response. The compounds accumulate during inflammation and can directly activate proinflammatory signalling pathways, including the induction of cytokines such as IL-6 and IL-8, and the activation of mitogen-activated protein kinase (MAPK) and nuclear factor kappa-light-chain-enhancer of activated B cells (NF-κB) pathways in immune and muscle cells [119]. Potential interference through hepatitis B vaccination at or near birth, around the time when the bloodspots were collected, is a possible explanation; however, it is assumed that bloodspots would have been collected pre-HB vaccination, or if infants were unwell, then collection would have been delayed appropriately. The results could suggest a relationship to infection/inflammation; however, the levels of acylcarnitines observed in SIDS were less than those in sepsis deaths or genetic disorders, which could reflect true pathophysiological differences or bias through uncertainty with SIDS diagnoses, given the retrospective nature of the study, which examined data from the years 2005 to 2011.

Maternofetal infection is suspected to be a risk factor for SIDS and could plausibly explain the acylcarnitine findings (i.e., maternal and/or fetal origin of the metabolite). The prevailing triple-risk model for SIDS posits that infection, including maternofetal infection, may act as an exogenous trigger in infants with underlying vulnerabilities, but evidence for a direct role of maternofetal infection is limited and complicated by the multifactorial nature of SIDS [1].

In infants (especially preterm), inflammation can induce apneic episodes [120,121], which carry the risk of SUD. It is now becoming apparent that intermittent hypoxia in preterm babies produces an inflammatory response in much the same way as it does in adults. This phenomenon of infant apnea and intermittent hypoxia has been an area of intense interest for mainstream SIDS researchers, wherein the focus has been on respiratory homeostasis rather than on inflammation. The natural progression of this work should include the inflammatory effects of vaccination as discussed above.

Immune paralysis (immunoparalysis)

Vaccination, in addition to causing inflammation, can result in other forms of immunotoxicity such as immunoparalysis, which is an acquired immune suppression marked by decreased innate and adaptive responses, often seen in critical illness or sepsis. Experimental and clinical data indicate that some vaccines, particularly those containing strong adjuvants or administered in high antigenic loads, can induce transient or persistent immunosuppressive effects [122-124]. For example, randomized trials of the DTP vaccine have been shown to induce immunotolerance, with long-term inhibition of monocyte-derived cytokine responses and diminution of T-cell reactivity to unrelated antigens, a phenomenon partially reversed by administration of Bacillus Calmette-Guérin (BCG) vaccine [125].

In mice, non-specific immunization with high antigen loads leads to adaptive immunosuppression as occurs in sepsis, lowering antibody responses and T-cell proliferation, partly due to the effects of regulatory T cells (Treg) [123]. Additionally, vaccine adjuvant-induced inflammation recruits inflammatory monocytes that suppress T-cell responses, acting as a counter-regulatory mechanism to limit excessive immune activation [124].

While these immunosuppressive effects are generally transient and rarely result in clinically significant immunoparalysis in healthy individuals, they highlight the complex immunomodulatory potential of vaccines beyond their intended antigen-specific effects. The clinical relevance of vaccine-induced immunoparalysis remains under investigation, but current evidence supports the possibility of immunosuppressive sequelae, particularly in the context of repeated or high-dose immunization, or in individuals with underlying immune dysregulation [126-130]. Infants, particularly those born prematurely, could be subject to vaccine-induced immunoparalysis, especially when exposed to high antigen loads provided through polyvalent vaccines given under the conditions imposed by national childhood immunization schedules, wherein up to 69 vaccine targets (or more, especially for Aboriginal infants) may be given in the first year of life [64].

Mechanisms Involved in Immunoparalysis

The mechanisms by which immunoparalysis occurs are complex and involve several different processes that fall into three main categories: immune cell death, anergy, and an anti-inflammatory state [130]. Recently, it has been shown that immune paralysis involves several processes, including immune-metabolic dysfunction, which adversely affects immune cell energy metabolism, transcriptomic changes affecting gene expression, and epigenetic effects. How sepsis, or vaccines (with their adjuvants), could affect these processes remains to be determined and is a subject requiring urgent investigation.

Sufficient evidence shows that immune paralysis does occur in infancy during or after a severe systemic inflammatory insult such as sepsis (as may occur in critically unwell babies in intensive care) and is characterized by a marked diminution in innate and adaptive immune responses accompanied by decreased expression of monocyte human leukocyte antigen-DR (HLA-DR) and diminution of in vitro levels of the proinflammatory cytokines TNF-α and IL-6 [130-132]. Affected infants are at risk of nosocomial infections and further sepsis. The pathophysiology involves a compensatory anti-inflammatory response following the initial proinflammatory insult and leads to downregulation of leukocyte function and altered gene expression that dampens immune activation [133]. Genomic profiling in affected infants has revealed increased expression of genes that actively suppress immune responses and limit expression of genes involved in immune activation and regulation, particularly those related to antigen handling and regulation of IL-6 production [129].

Weiss et al. [130] were the first to describe mitochondrial dysfunction as an important player in immune paralysis, the evidence of which was derived from both animal models and human cases. The processes involved are multifactorial and affect both the innate and adaptive arms of the immune system. Mitochondrial dysfunction in peripheral blood mononuclear cells contributes to immune paralysis through disruption of cellular energy metabolism and immune competence [130].

Expansion of myeloid-derived suppressor cells, mainly polymorphonuclear subsets generated via the Programmed Cell Death Protein 1 and Programmed Cell Death Ligand (PD-1/PD-L1) pathway, further suppresses T-cell activation and cytokine production, perpetuating immune paralysis [131].

In infants, the immaturity of their immune system plus the presence of transplacental maternal antibodies further modulate immune responses, so immune paralysis, as described above, is a distinct ‘acquired’ phenomenon, most often triggered by severe systemic inflammation or infection [131-133]. It remains unclear whether maternal antibodies contribute to immune paralysis.

Immune cell death or apoptosis, as mentioned, is a key process involved in immune paralysis. Sepsis-induced apoptosis in human subjects results in progressive removal of CD4 T+-cells, CD8 T-cells, B-cells, natural killer (NK) cells, and follicular dendritic cells. Apoptosis occurs through two pathways: the death-receptor and the mitochondrial-mediated pathway. Macrophages and dendritic cells that engulf apoptotic cell debris upregulate anti-inflammatory cytokines, notably IL-10 by Treg cells, as part of the immune-silencing response to apoptotic cell clearance [134]. Alternatively, a state of anergy may extend immunosuppressive effects through decreased co-expression of monocyte CD14/HLA-DR.

Given that hyperimmunization is known to cause immune paralysis, it is reasonable to suppose that the infant immunization schedule, with multiple antigen exposures at one time (mimicking hyperimmunization), could also be responsible for this adverse outcome.

The consequences of hyperimmunization or infection may include the production of a “cytokine storm” associated with the systemic inflammatory response syndrome (SIRS) [135,136]. Often this is followed by compensatory anti-inflammatory response syndrome (CARS), which is the immune system's counter-inflammatory response to sepsis, a mechanism to restore homeostasis following SIRS. While SIRS is a proinflammatory response to fight infection, CARS is a systemic immunosuppression that can increase susceptibility to secondary infections and lead to poor clinical outcomes, including sepsis [135-137].

Considering overwhelming CARS, immune paralysis may develop through the processes discussed above (immune cell apoptosis and functional impairment of lymphocytes and phagocytes). The process is marked by increased anti-inflammatory and decreased pro-inflammatory cytokine production [135-138]. The role of Treg lymphocytes in immune suppression is becoming better understood and shows that mitochondrial and lysosomal signalling orchestrate the metabolic and functional fitness of Treg cells [139,140].

The anti-inflammatory response is known to overshoot. This predisposes the host to bacterial infection (and risk of sepsis), and/or reactivation of latent viruses [141,142]. Studies show that monocyte deactivation and impaired immune responses are present in CARS [141]. It can be concluded that, based on immune cell phenotypes, fatal sepsis bears the hallmarks of immunosuppression [143].

Ferrante et al. [144] reported another potential contributor to immune paralysis: downregulation of myeloid differentiation primary response gene 88 (Myd88) in cerebral tissue from SIDS. The authors found downregulation of MyD88 in brain tissue, and downregulation of CCL3 and UNC13 in liver tissue of SIDS cases. These genes are involved in immune system function and inflammatory processes. Their findings suggest a subset of SIDS cases may have impaired or dysregulated immune responses. This supports the hypothesis that immunodeficiency or altered inflammatory gene expression may contribute to SIDS pathogenesis. The study adds to the growing body of evidence implicating immune system vulnerability as a potential intrinsic risk factor for SIDS, rather than infection alone being the trigger [1,16]. On the other hand, the paper by Korzun et al [117] hypothesized that lipids in LNPs used in mRNA vaccines could activate Toll-like receptor-4 (TLR4) signalling via MyD88 and Toll/interleukin-1 receptor domain-containing adaptor-inducing interferon-β adaptors (TRIF adaptors), thereby propagating LNP-associated reactogenicity and thence contribute to inflammatory load [117].

As immune paralysis is an established entity in infancy, it is appropriate for this review to examine if there is a putative role of this immunopathology in SUDI and SIDS. And by extension, the review explores why immune paralysis could be important in the context of infant immunization and SIDS/SUDI.

Evidence of a sepsis-like process in SIDS

While sepsis is an obvious sequela of immune paralysis, could this form of immunopathology arise from vaccination and hyperimmunization? Previous studies have demonstrated positive evidence for a sepsis-like process occurring in susceptible babies who die of SIDS (thus opening the possibility that sepsis is a consequence of immune paralysis). The evidence for a sepsis-like process in SIDS includes the following findings: raised proinflammatory cytokines in SIDS [14,20,145-148], bacterial toxins in SIDS tissues [11,149], isolation of highly pathogenic bacteria (Staphylococcus aureus, Escherichia coli) in normally sterile sites [150-152], elevated fibrin degradation products in sera from SIDS cases [153], shock-like physiological evidence of tachycardia followed by profound bradycardia occurring before cessation of breathing in cases of SIDS captured on memory monitors [154,155], and biochemical/clinical chemistry findings of McGaffey that reflected evidence of sepsis. Her work showed elevated brain and CSF lactate in conjunction with low CSF pH in cohorts of SIDS cases [156-160]. Other indicators included organ weight changes (heavy, wet lungs, large brain and liver) compatible with sepsis, with enlarged thymus reflecting immunological perturbation [161-164], and intrathoracic petechial hemorrhages, possibly the consequence of sepsis-related immune-mediated vasculopathy causing capillary leakage through basement membrane disruption [165,166], and shock-like diaphragm histopathology [167,168] with contraction band necrosis and muscle fibre changes frequently reported but not universally observed.

Clues from the thymus

In neonates, respiratory viral infections may affect the thymus (the key organ in adaptive immune system development) by causing a series of morphological changes. Early in the infection, the thymus becomes edematous and enlarges, and then thymic reticular epithelial cell collapse follows. Later in the infection, thymic shrinkage (involution) occurs with volume loss in the cortex and medulla, followed by fibrosis [169]. Based on these neonatal findings, the overall significance of thymic enlargement in SIDS suggests that most mortal events occur relatively soon after viral infection (before the process of involution), while there are also cases in which the thymus is smaller than average, suggesting a longer duration of infection with possible downstream effects from sepsis and CARS [141].

According to MacFarlane [14], the role of the thymus is to modulate the development of the adaptive immune system. Organ weight studies have shown SIDS babies have generally heavier thymuses than controls [167-169]. This was confirmed by Qu and colleagues [170] and supported the aetiological involvement of the organ in SIDS. These authors also found increased concentrations of several pro-inflammatory cytokines in SIDS compared to infants who had no infectious component in their death. The level of some cytokines in SIDS was within the range (but generally less) than that of control infants who died with severe infection (sepsis or pneumonia). As most of the cytokines promote thymic (and T-cell) development, this suggests that there may have been an underlying moderate infection in these infants and supports the idea of subclinical infection proposed by Goldwater et al. [162]. Moreover, these data highlight the key observation that these thymic differences were unique to SIDS infants less than five months of age, which coincides with the timing of the peak incidence of SIDS (two to six months) and the beginning of the immunization schedule.

According to MacFarlane [14], the data indicate a subclinical activation of the immune system in the youngest SIDS infants, indicating a lack of age-dependent thymus maturation. MacFarlane also proposed that SIDS involve a “repression of normal immune system development” but also proposed that “the cytokine profiles and thymic weights were already elevated in the youngest SIDS cases (less than five months of age), suggesting a prematurely developed or over-reactive immune system” [14].

Thus, these characteristics of the thymus in the younger (less than five months) SIDS infants commenced higher than in control infants, perhaps masking normal postnatal maturational processes. Both ideas support and do not negatively impact the overwhelming evidence of infection (or foreign mediators of inflammation) playing a role in SIDS. The thymic findings should encourage deeper analysis of thymic and immune abnormalities in SIDS and should promote intensive study of immune system development in relation to critical stages of vulnerability in SIDS and in healthy subjects.

Animal studies

Animal studies may provide useful information by comparing corresponding critical stages of vulnerability (the prominent component of the triple risk hypothesis) as a model for SIDS. For instance, the infant rat model of SIDS examines this and reveals a similar heightened vulnerability to mild proinflammatory challenges observed in SIDS [171-173]. The findings of these studies point strongly to an immunopathology resulting in shock, and their findings do not diminish the idea of subclinical infection being part of the underlying mechanism. Thus, the door remains open to the idea that a form of 'pseudo-infection' (i.e., vaccination or hyperimmunization) acts as a precipitant.

Bajanowski et al. [174] studied 50 cases of SIDS. Lymphoid tissues, including cervical, paratracheal, and lung hilar lymph nodes, thymus, and spleen, were examined histologically and immunohistochemically (using anti-CD 20, 21, 45RO staining). Pronounced lymphodepletion of the thymus (assumed to indicate prolonged T-cell system stimulation) was demonstrated. No cases had defects of the T- or B-cell system [174]. The lymph node and thymic findings were compatible with subclinical infection as an underlying mechanism in SIDS pathogenesis.

Nouri et al. [96], while omitting the thymus, followed peripheral blood cell gene expression following immunization at two months of age. The setting of the study could represent a situation of hyperimmunization with infants receiving their second HepB dose and their initial doses of DTaP, Hib, IPV, pneumococcal conjugate (PCV13), and live attenuated rotavirus (RV) vaccines. All (21 antigens) except the oral RV vaccine were administered by injection. On day seven post-vaccination, the authors observed a substantial increase in the expression of three IFN-related gene modules, a monocyte gene module, and several inflammation-associated modules [96]. Extreme heterogeneity of these was observed, with individuals showing over- or under-expression of key genes. Such extreme responses, particularly those involving genes contributing to inflammation, could contribute to immunopathological adverse outcomes.

Limitations

Decades of research have shown that infection and inflammation are consistent, common features of SIDS, and that immunization induces inflammatory cytokine changes similar to those observed during infections, suggesting two common pathways to inflammation. Blood-Siegfried (2009) [5] reviewed infection and inflammation in SIDS and concluded that a high proportion of cases less than six months of age had these features, but did not link inflammation with immunizations. Mainstream SIDS research has shown that brainstem pathology, central to the triple risk hypothesis, occurs in about 50% of cases of SIDS based on Naeye’s [175] finding of brainstem astrogliosis. Decades of research have added brainstem neuronal apoptosis and microglial activation to astrogliosis as markers of neuroinflammation [176]. These changes are purported to also underlie homeostatic failure of respiration, cardiac function, or arousal, according to the triple risk hypothesis. But equally, they are most likely the result of infection and related proinflammatory cytokines, given that the observed inflammation (together with its cause) must precede supposed effects on homeostasis.

When vaccines are given simultaneously in multiples or in high doses, the phenomenon of immune paralysis is a known sequela. The immature immune system of young infants (together with lack of breastmilk and/or transplacental antibodies, etc.) increases vulnerability to infection and sepsis and can lead to immune paralysis following a “cytokine storm,” so, theoretically, infection, sepsis, and vaccines could produce this outcome. The key limitation of this paper is insufficient knowledge concerning the effects of immunization in this process. However, the above detailed discussion has exposed a “signal” representing a possible relationship between vaccines and SIDS, and this calls for further investigation of infant immunization, given the schedule provides vaccinations in a context akin to hyperimmunization. Results of better-designed future studies are awaited.

The level of certainty that vaccines do not cause SIDS is also debatable, as discussed above. The studies show that those who do or do not receive vaccines are likely to differ in many ways, including the subsequent risk of early death that is independent of vaccination. Other biases include subject and control selection, the selective loss of vaccination records for children who die, etc. [177].

Moreover, infants and children who receive vaccines are likely to differ in many ways from those who do not (the “healthy vaccinee effect”), which is a problem for observational studies. Vaccine uptake patterns for different populations and non-specific effects introduce further biases [177-184]. Socio-economic status, amongst other factors, also carries additional mortality risk biases and may act as a potential confounder for possible associations between vaccination and mortality. Examples include poverty (and its increased risk of infectious disease). These socioeconomic factors, such as low parental education and distance from a health centre, may also play a role [178]. Many socioeconomic aspects are associated with low or delayed vaccine uptake and tend to be associated with high mortality [177]. On the other hand, the link between high socio-economic status and high vaccination rates is expected to reduce mortality rate ratios when comparing those being vaccinated and those not undergoing vaccination. Moreover, the collection of data on such factors and their use in adjusting analyses requires ultimate care.

The use of statistical methods, including case-control and self-controlled case series designs, can usually robustly assess associations between vaccines and rare outcomes such as SIDS, but cannot fully rule out associations due to confounding and bias, for extremely rare events as discussed above [69,74]. These methods have been used in large studies and meta-analyses, which consistently show no increased risk, and in some, a reduced risk of SIDS following vaccination, even when examining short post-vaccination intervals. However, these findings can mask or exaggerate true associations [69,74,185]. Goldman and Cheng [185] introduced another relevant issue regarding how CYP450 enzyme immaturity and variability may influence the metabolism of vaccine excipients and their relevance to immune and safety outcomes in infancy. The authors state that current vaccination schedules, which do not fully account for individual variability in metabolism, overlook the impact of polymorphisms in enzymes like CYP2D6, CYP2C19, and CYP3A4. This may lead to prolonged exposure to potentially toxic excipients or altered immune responses in infants with poor metabolizer phenotypes [185]. One of their main concerns related to the determination of the diagnosis owing to the variability of autopsy protocols, as this can obscure important factors affecting statistical analyses, because some deaths misclassified as SIDS obscure vaccine-related outcomes. Based on the above points, the authors strongly supported further study of potential links between vaccines and SIDS.

Surveillance systems and observational studies are essential for ongoing safety monitoring, but spontaneous reporting databases (e.g., VAERS) cannot establish causality due to lack of denominators and reporting biases. Because VAERS does not capture the total number of vaccine doses administered (denominator data), it cannot calculate incidence rates or relative risks and thus cannot determine whether a vaccine caused an adverse event [84,186]. While current evidence strongly supports vaccine safety regarding SIDS [1], the American Academy of Pediatrics notes that statistical methods, including those used in population-based studies and passive surveillance systems, cannot definitively exclude extremely rare associations due to limitations such as underreporting, lack of denominator data, and confounding factors [1]. Also, biological plausibility and consistent findings across diverse populations are necessary for causal inference [1].

Possible rare adverse outcomes arising from immunizations raise other considerations and include imperceivable harms which may be hidden by productivity pressures, complacency, social influences, and vested interests. Thus, the reader might even consider that normalization of deviance could operate with attitudes towards immunization safety. Vaccination programs must be cognizant of these influences.

Future direction

**Figure 2:**
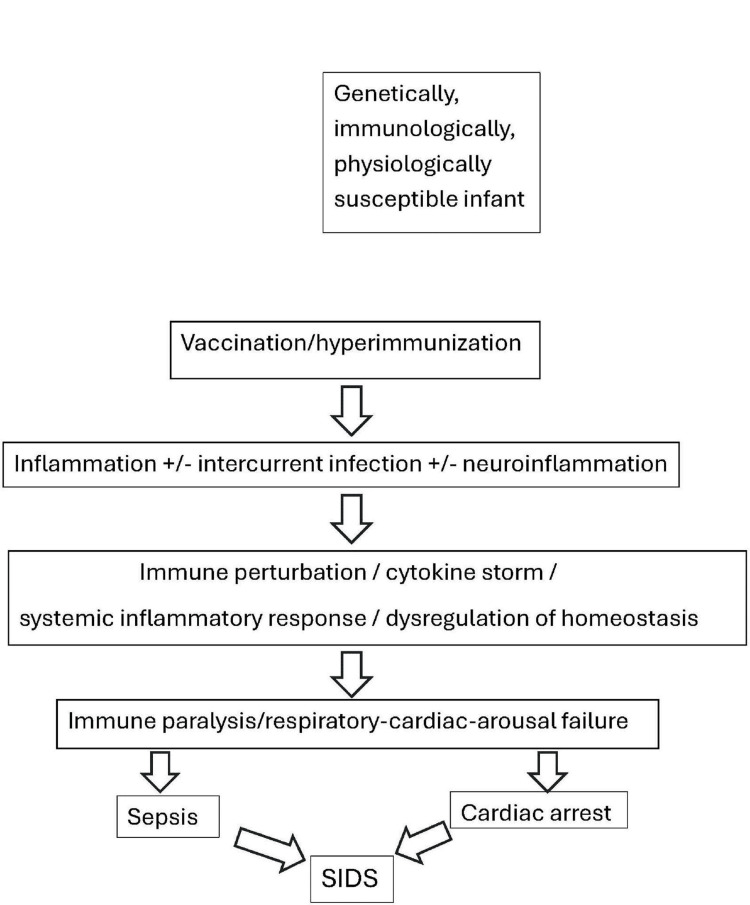
Graphical summarizing the major aspects considered in the review SIDS: Sudden infant death syndrome. Image credit: Created by the author using Microsoft Word (Microsoft Corp., Redmond, WA, USA).

While this review offers a biologically plausible hypothesis to explain an inflammation-related subgroup of unexplained sudden infant deaths (summarized in Figure 2), it should be read in the context of encouraging further research in this area to provide definitive answers and to encourage the development of safer vaccines.

While it is encouraging to see active development of mucosal vaccines, should we be concerned about the reactogenicity of these vaccines in the context of hyperimmunization and immune paralysis? Current mucosal vaccines (intranasal, inhaled, and oral) are generally less reactogenic than parenterally administered vaccines. Mucosal vaccines tend to cause fewer systemic adverse reactions (e.g., fever, myalgia, malaise). Systematic reviews and meta-analyses of mucosal vaccines for respiratory pathogens (including COVID-19 and influenza) indicate that their safety profile is comparable to or better than intramuscular vaccines, with adverse events typically mild and transient. No significant increase in serious adverse events has been observed with mucosal vaccines in large clinical trials [187]. However, reactogenicity can vary depending on the vaccine platform (live attenuated, inactivated, or vector-based), the mucosal route, and the use of adjuvants [188,189]. It will take time to understand if multiple mucosal vaccines can be given together and avoid suspected rare hyperimmunization effects observed with parenteral vaccines.

The apparent lower reactogenicity of mucosal vaccines, and other advantages [188,190] should encourage their development, especially in view of the issues surrounding the high number of vaccines and needles given in an infant’s first year of life. It is predicted that mucosal vaccines will eventually overtake those given parenterally.

It must be stressed that this review in not intended to discourage parents from having their babies immunized. Avoidance of risk factors (smoking, risky sleeping surfaces, prone sleep position, overwrapping, co-sleeping, etc.) and encouraging breastfeeding and perhaps postponing immunization if there is evidence of an infection would seem sensible. Maternal immunization during pregnancy should be encouraged to prevent Respiratory Syncytial Virus (RSV) in infancy [191]. Based on general principles of prevention of infectious disease, encouragement of hand hygiene and the use of clean bed linen could be helpful in reducing risk.

## Conclusions

Through the decades of research into the cause(s) of SIDS, the overwhelming weight of evidence indicates that inflammation is a strong causal contender. Two main mechanisms may lead to inflammation: infection and vaccination, singly or in combination. Inflammation can lead to immune paralysis, and then to shock and unexpected death. Shock is a stealth killer and is notoriously difficult to diagnose.

Inflammation likely underlies the mechanisms espoused by mainstream researchers (disruption of homeostasis of breathing, cardiac function, and arousal). Both points of view have been discussed in this rapid review. In many respects, the evidence presented regarding infection and/or inflammation stands well supported by a broad swathe of evidence. It seems apparent that both the triple-risk hypothesis and the infection/vaccination/inflammation hypothesis can coexist, but, on balance of probability, the former requires the latter for inflammation-related SIDS to occur. The review has not discussed the effect of anemia on the occurrence of SIDS; there are interesting physiological, immunological, and developmental interactions relating to infection and immunization in the anemic infant that require full exploration. These interactions are complex and require a separate review for proper discussion. 

Whether or not vaccinations, as they are currently administered, are a significant player in inflammation-related SIDS will be clarified after the performance by bias-free research of well-designed studies that control for all possible factors affecting risk for SIDS and have sufficient statistical power to provide meaningful results. Mucosal immunization may prove a safer and immunologically better alternative to parenterally administered vaccines because of its several advantages in terms of induction of T-cell response and the avoidance of the use of needles.
